# Supplementary material for: Associations between ethnicity and persistent physical and mental health symptoms experienced as part of ongoing symptomatic COVID-19
Source: PLoS One. 2024 Oct 31;19(10):e0312719. doi: 10.1371/journal.pone.0312719 (PMC11527325; doi:10.1371/journal.pone.0312719)
Supplement: S1 Table — (DOCX) [file pone.0312719.s002.docx]

**Supplementary Table 1: Comparison between observed characteristics of participants with known and unknown ethnicities**

|  | **Ethnic group known**  **(n=902)** | **Ethnic group unknown**  **(n=84)** | **p-value** |
| --- | --- | --- | --- |
| **Demographics and co-morbidities** | | | |
| Age (years) | 61.00  (50.75 – 73.00) | 60.00  (46.25 – 73.75) | 0.67 |
| Sex (female) (%) | 364 (40) | 28 (33) | 0.21 |
| Deprivation decile* | 5.00  (3.00 – 7.00) | 5.00  (3.00 – 7.00) | 0.52 |
| Body mass index (kg/m^2^) | 26.99  (23.95 – 31.10) | 28.23  (24.98 – 30.76) | 0.38 |
| Hypertension (%) | 399 (44) | 29 (35) | 0.09 |
| Diabetes (%) | 185 (21) | 7 (8) | **0.007** |
| Any cardiac disease (%) | 206 (23) | 15 (18) | 0.30 |
| Cerebrovascular disease (%) | 72 (8) | 6 (7) | 0.79 |
| Chronic lung disease (%) | 162 (18) | 12 (14) | 0.40 |
| Ever smoked (%) | 293/862 (34) | 28/79 (35) | 0.79 |
| Chronic kidney disease (%) | 116 (13) | 5 (6) | 0.07 |
| Immunosuppressed due to cancer or autoimmune disease (%) | 134 (15) | 7 (8) | 0.10 |
| Mental health condition (%) | 128 (14) | 12 (14) | 0.98 |
| Admission clinical frailty score | 3.00  (2.00 – 4.00) | 3.00  (2.00 – 4.00) | 0.11 |
| **COVID-19 characteristics** | | | |
| Wave: wild-type  Wave: alpha  Wave: delta (%) | 213 (24)  609 (68)  80 (9) | 48 (57)  28 (33)  8 (9.5) | **<0.001** |
| Vaccinated on admission (%) | 34/254 (13) | 2/10 (20) | 0.55 |
| Admission number of symptoms^†^ | 3.00  (2.00 – 4.00) | 3.00  (2.00 – 5.00) | **0.03** |
| National Earning Warning System 2 Score (NEWS2) | 4.00  (2.00 – 6.00) | 5.00  (3.00 – 7.00) | **0.006** |
| Full escalation (%) | 738/808 (91) | 70/79 (89) | 0.42 |
| Severe infection (%) | 168/814 (21) | 18/82 (22) | 0.78 |
| Steroids prescribed (%) | 433/617 (70) | 21/67 (31) | **<0.001** |
| Other drugs e.g. tocilizumab, remdesivir prescribed (%) | 187/565 (33) | 13/70 (17) | **0.01** |
| **Outcomes at follow-up** | | | |
| Respiratory symptoms^§^ (%) | 460/880 (52) | 41/83 (49) | 0.62 |
| Fatigue (%) | 528/878 (60) | 44/79 (56) | 0.44 |
| Poor sleep quality (%) | 315/866 (36) | 32/80 (40) | 0.52 |
| Number of symptoms at follow-up^¶^ | 0.00  (0.00 – 1.00) | 0.00  (0.00 – 2.00) | 0.65 |
| Affected mental health (%) | 112 (12) | 8 (10) | 0.44 |
| Inability to return to work (%) | 179/429 (42) | 14/43 (33) | 0.24 |

*Data are presented as median (interquartile range) for non-parametric data and proportions (in percentages) for categorical data.
*Lower deciles indicate higher deprivation.*^†^*Number of symptoms out of 19 and included: cough, shortness of breath, sore throat, rhinitis, fever, chills, fatigue, myalgia, headache, anorexia, anosmia, loss of taste, diarrhoea, abdominal pain, chest pain, chest tightness, confusion, peripheral oedema and focal weakness.*

^§^*Presence of cough and/or shortness of breath.*

^¶^*Number of symptoms out of 10 and included: chest pain, chest tightness, myalgia, anosmia, anorexia, abdominal pain, diarrhoea, leg swelling, confusion and focal weakness.*
