# Supplementary material for: Associations between ethnicity and persistent physical and mental health symptoms experienced as part of ongoing symptomatic COVID-19
Source: PLoS One. 2024 Oct 31;19(10):e0312719. doi: 10.1371/journal.pone.0312719 (PMC11527325; doi:10.1371/journal.pone.0312719)
Supplement: S2 Table — (DOCX) [file pone.0312719.s003.docx]

**Supplementary Table 2: Follow-up outcomes of adults from different ethnic groups adjusted for propensity score**

|  | **Adjusted OR**  **(95% CI)** ^‡^ | **p-value** | **Propensity score adjusted OR**  **(95% CI)** ^‡^ | **p-value** |
| --- | --- | --- | --- | --- |
| **Respiratory symptoms***  Asian  Black  Mixed  White  Other | 0.62 (0.37 – 1.06)  **0.49 (0.25 – 0.96)**  0.51 (0.10 – 2.47)  1.00 (ref)  1.05 (0.57 – 1.93) | 0.08  **0.04**  0.40  -  0.87 | 0.58 (0.34 – 0.99)  **0.46 (0.23** – **0.93)**  0.51 (0.11 – 2.51)  1.00 (ref)  0.97 (0.53 – 1.80) | 0.05  **0.03**  0.42  -  0.93 |
| **Fatigue**  Asian  Black  Mixed  White  Other | 0.95 (0.55 – 1.64)  0.63 (0.32 – 1.24)  3.79 (0.44 – 33.01)  1.00 (ref)  0.76 (0.42 – 1.38) | 0.86  0.18  0.23  -  0.37 | 0.93 (0.53 – 1.60)  0.61 (0.31 – 1.22)  3.81 (0.43 – 33.66)  1.00 (ref)  0.73 (0.40 – 1.33) | 0.78  0.16  0.23  -  0.30 |
| **Poor sleep quality**  Asian  Black  Mixed  White  Other | 0.74 (0.43 – 1.28)  0.56 (0.27 – 1.13)  0.95 (0.18 – 4.97)  1.00 (ref)  1.21 (0.67 – 2.19) | 0.28  0.10  0.95  -  0.53 | 0.75 (0.44 – 1.31)  0.53 (0.26 – 1.10)  1.06 (0.20 – 5.53)  1.00 (ref)  1.12 (0.66 – 2.18) | 0.31  0.09  0.94  -  0.56 |
| **Number of symptoms at follow-up^†^**  Asian  Black  Mixed  White  Other | 0.74 (0.50 – 1.10)  **0.68 (0.34 – 0.99)**  1.26 (0.42 – 3.80)  1.00 (ref)  0.75 (0.48 – 1.19) | 0.14  **0.046**  0.68  -  0.22 | 0.74 (0.50-1.10)  **0.55 (0.32 – 0.94)**  1.31 (0.43 – 4.00)  1.00 (ref)  0.73 (0.46 – 1.16) | 0.140  **0.03**  0.64  **-**  0.19 |
| **Affected mental health**  Asian  Black  Mixed  White  Other | 0.84 (0.38 – 1.84)  1.44 (0.56 – 3.70)  2.98 (0.46 – 19.28)  1.00 (ref)  0.78 (0.30 – 2.05) | 0.66  0.45  0.25  -  0.61 | 0.92 (0.41 – 2.03)  1.31 (0.49 – 3.47)  3.13 (0.48 – 20.76)  1.00 (ref)  0.83 (0.32 – 2.20) | 0.83  0.59  0.24  -  0.71 |
| **Inability to return to work**  Asian  Black  Mixed  White  Other | 1.16 (0.55 – 2.45)  0.62 (0.22 – 1.70)  0.69 (0.09 – 5.49)  1.00 (ref)  0.91 (0.38 – 2.17) | 0.69  0.35  0.73  -  0.82 | 1.18 (0.56 – 2.50)  0.58 (0.20 – 1.68)  0.66 (0.08 – 5.37)  1.00 (ref)  0.87 (0.36 – 2.10) | 0.67  0.32  0.70  -  0.75 |
