# Supplementary material for: Associations between ethnicity and persistent physical and mental health symptoms experienced as part of ongoing symptomatic COVID-19
Source: PLoS One. 2024 Oct 31;19(10):e0312719. doi: 10.1371/journal.pone.0312719 (PMC11527325; doi:10.1371/journal.pone.0312719)
Supplement: S3 Table — (DOCX) [file pone.0312719.s004.docx]

**Supplementary Table 3: Variables associated with follow-up outcomes**

| **Outcome: Respiratory symptoms (logistic regression)** | | |
| --- | --- | --- |
| **Predictor** | **Odds ratio (95% CI)** | **p-value** |
| Intercept | 4.01 (0.42 – 38.68) | 0.23 |
| Age | 1.00 (0.98 – 1.01) | 0.60 |
| Index of deprivation | 1.00 (0.91 – 1.10) | 0.98 |
| Body mass index (BMI) | 1.02 (0.98 – 1.06) | 0.31 |
| Clinical frailty score | 1.00 (0.85 – 1.18) | 0.98 |
| Time to follow-up | 0.99 (0.99 – 1.00) | 0.10 |
| Sex (male) | 0.73 (0.47 – 1.12) | 0.15 |
| Hypertension (absence) | 0.96 (0.61 – 1.52) | 0.87 |
| Diabetes (absence) | 1.00 (0.55 – 1.79) | 0.99 |
| Smoking status (never-smoker) | 0.63 (0.40 – 0.99) | **0.04** |
| Wave: wild-type  Wave: alpha | 0.82 (0.10 – 6.52)  1.73 (0.66 – 4.55) | 0.85  0.27 |
| Acute COVID-19 severity (moderate) | 0.56 (0.33 – 0.95) | **0.03** |
| Treatment with steroids | 1.17 (0.17 – 8.12) | 0.88 |
| **Outcome: Fatigue (logistic regression)** | | |
| **Predictor** | **Odds ratio (95% CI)** | **p-value** |
| Intercept | 1.11 (0.12 – 10.27) | 0.93 |
| Age | 1.00 (0.98 – 1.01) | 0.80 |
| Index of deprivation | 1.05 (0.95 – 1.15) | 0.33 |
| Body mass index (BMI) | 1.01 (0.97 – 1.04) | 0.81 |
| Clinical frailty score | 0.99 (0.84 – 1.17) | 0.93 |
| Time to follow-up | 1.00 (0.99 – 1.00) | 0.85 |
| Sex (male) | 0.81 (0.52 – 1.25) | 0.34 |
| Hypertension (absence) | 0.80 (0.51 – 1.27) | 0.35 |
| Diabetes (absence) | 1.20 (0.67 – 2.15) | 0.54 |
| Smoking status (never-smoker) | 0.95 (0.61 – 1.49) | 0.83 |
| Wave: wild-type  Wave: alpha | 8.11 (0.71 – 93.0)  2.71 (1.06 – 6.90) | 0.09  **0.04** |
| Acute COVID-19 severity (moderate) | 0.67 (0.39 – 1.13) | 0.13 |
| Treatment with steroids | 0.22 (0.02 – 2.34) | 0.21 |
| **Outcome: Poor sleep quality (logistic regression)** | | |
| **Predictor** | **Odds ratio (95% CI)** | **p-value** |
| Intercept | 0.57 (0.00 – 1.00) | 0.05 |
| Age | 0.99 (0.97 – 1.01) | 0.18 |
| Index of deprivation | 0.96 (0.88 – 1.05) | 0.40 |
| Body mass index (BMI) | 1.04 (1.00 – 1.08) | 0.45 |
| Clinical frailty score | 1.07 (0.90 – 1.26) | 0.45 |
| Time to follow-up | 1.00 (1.00 – 1.01) | 0.36 |
| Sex (male) | 1.05 (0.68 – 1.62) | 0.84 |
| Hypertension (absence) | 0.63 (0.40 – 0.99) | **0.05** |
| Diabetes (absence) | 1.04 (0.57 – 1.88) | 0.90 |
| Smoking status (never-smoker) | 0.74 (0.48 – 1.14) | 0.17 |
| Wave: wild-type  Wave: alpha | N.D.  11.85 (1.50 – 93.16) | N.D.  **0.02** |
| Acute COVID-19 severity (moderate) | 0.92 (0.56 – 1.52) | 0.75 |
| **Outcome: Number of symptoms at follow-up (negative binomial regression)** | | |
| **Predictor** | **Count ratio (95% CI)** | **p-value** |
| Intercept | 0.27 (0.05 – 1.62) | 0.15 |
| Age | 0.99 (0.98 – 1.00) | 0.21 |
| Index of deprivation | 0.98 (0.91 – 1.05) | 0.53 |
| Body mass index (BMI) | 1.02 (1.00 – 1.05) | 0.09 |
| Clinical frailty score | 1.06 (0.95 – 1.20) | 0.30 |
| Time to follow-up | 1.00 (1.00 – 1.00) | 0.84 |
| Sex (male) | 0.77 (0.56 – 1.05) | 0.10 |
| Hypertension (absence) | 0.83 (0.60 – 1.16) | 0.27 |
| Diabetes (absence) | 0.93 (0.61 – 1.43) | 0.75 |
| Smoking status (never-smoker) | 0.86 (0.63 – 1.18) | 0.35 |
| Wave: wild-type  Wave: alpha | 2.84 (0.65 – 12.46)  **4.13 (1.47 – 11.60)** | 0.17  **0.01** |
| Acute COVID-19 severity (moderate) | 0.94 (0.66 – 1.36) | 0.76 |
| Treatment with steroids | 1.59 (0.54 – 4.72) | 0.41 |
| **Outcome: Affected mental health (logistic regression)** | | |
| **Predictor** | **Odds ratio (95% CI)** | **p-value** |
| Intercept | 0.96 (0.04 – 23.77) | 0.98 |
| Age | 1.92 (0.99 – 1.05) | 0.14 |
| Index of deprivation | 0.97 (0.85 – 1.11) | 0.66 |
| Body mass index (BMI) | 0.98 (0.93 – 1.04) | 0.47 |
| Clinical frailty score | 0.85 (0.65 – 1.10) | 0.21 |
| Time to follow-up | 0.99 (0.98 – 1.00) | 0.21 |
| Sex (male) | 0.99 (0.54 – 1.83) | 0.98 |
| Hypertension (absence) | 1.30 (0.68 – 2.51) | 0.43 |
| Diabetes (absence) | 0.81 (0.37 – 1.77) | 0.60 |
| Smoking status (never-smoker) | 0.83 (0.45 – 1.53) | 0.55 |
| Wave: wild-type  Wave: alpha | 0.29 (0.02 – 4.72)  0.39 (0.13 – 1.17) | 0.39  0.09 |
| Acute COVID-19 severity (moderate) | 0.87 (0.43 – 1.75) | 0.69 |
| Treatment with steroids | 0.66 (0.05 – 9.40) | 0.76 |
| **Outcome: Inability to return to work (logistic regression)** | | |
| **Predictor** | **Odds ratio (95% CI)** | **p-value** |
| Intercept | 5.50 (0.21 – 143.29) | 0.31 |
| Age | 1.00 (0.98 – 1.02) | 0.92 |
| Index of deprivation | 0.88 (0.77 – 1.01) | 0.06 |
| Body mass index (BMI) | 1.01 (0.95 – 1.06) | 0.85 |
| Clinical frailty score | 0.93 (0.75 – 1.16) | 0.52 |
| Time to follow-up | 1.00 (0.99 – 1.02) | 0.67 |
| Sex (male) | 0.77 (0.41 – 1.42) | 0.40 |
| Hypertension (absence) | 0.61 (0.33 – 1.13) | 0.11 |
| Diabetes (absence) | 0.65 (0.28 – 1.48) | 0.30 |
| Smoking status (never-smoker) | 0.66 (0.35 – 1.22) | 0.18 |
| Wave: wild-type  Wave: alpha | N.D  1.43 (0.34 – 6.05) | N.D.  0.64 |
| Acute COVID-19 severity (moderate) | 1.13 (0.57 – 2.24) | 0.73 |

*N.D. is not determined.*
